# Supplementary material for: A PDZ-RapGEF promotes synaptic development in Caenorhabditis elegans through a Rap/Rac signaling pathway
Source: Development. 2025 Aug 26;152(16):dev204678. doi: 10.1242/dev.204678 (PMC12448318; doi:10.1242/dev.204678)
Supplement: Supplementary information [file develop-152-204678-s1.pdf]

Table S1. *C. elegans* strains used in this study

Genotype	Reference	Strain
wild type (N2)		CGC257
rap-1(pk2082) IV	(Pellis-van Berkel et al., 2005)	TZ181
rap-2(gk11) V	From VC14 (Thompson et al., 2013)	SJC486
rac-2(ok326) IV	From VC126 (Lundquist et al., 2001)	SJC366
rac-2(gk281) IV	(Consortium, 2012)	VC583
ced-10(n1993) IV	From LE712 (Lundquist et al., 2001)	SJC202
Punc-129::mCherry::RAB-3(tauIs46)	(Zhou et al., 2017)	SJC182
pxf-1(gk955083) IV; Punc-129::mCherry::RAB-3(tauIs46)	(Lamb et al., 2022)	SJC324
Punc-129::mCherry::RAB-3(tauIs46); Prgef-1::pxf-1 cDNA (bluEx53)	This study	SJC698
pxf-1(gk955083) IV; Punc-129::mCherry::RAB-3(tauIs46); Prgef-1::pxf-1 cDNA (bluEx53)	This study	SJC632
Punc-129::mCherry::RAB-3(tauIs46); Prgef-1::pxf-1 cDNA (bluEx54)	This study	SJC699
pxf-1(gk955083) IV; Punc-129::mCherry::RAB-3(tauIs46); Prgef-1::pxf-1 cDNA (bluEx54)	This study	SJC633
Punc-129::mCherry::RAB-3(tauIs46); Punc-17b::pxf-1 cDNA (bluEx59)	This study	SJC702
pxf-1(gk955083) IV; Punc-129::mCherry::RAB-3(tauIs46); Punc-17b::pxf-1 cDNA (bluEx59)	This study	SJC598
Punc-129::mCherry::RAB-3(tauIs46); Punc-17b::pxf-1 cDNA (bluEx61)	This study	SJC682
pxf-1(gk955083) IV; Punc-129::mCherry::RAB-3(tauIs46); Punc-17b::pxf-1 cDNA (bluEx61)	This study	SJC599
rac-2(ok326) IV; Punc-129::mCherry::RAB-3(tauIs46)	This study	SJC373
rap-1(pk2082) IV; Punc-129::mCherry::RAB-3(tauIs46)	This study	SJC505
rap-2(gk11) V; Punc-129::mCherry::RAB-3(tauIs46)	This study	SJC470

Development • Supplementary information

pxf-1(gk955083) IV; rac-2(ok326) IV; Punc-129::mCherry::RAB-3(tauIs46)	This study	SJC439
tiam-1(tm1556) I; Punc-129::mCherry::RAB-3(tauIs46)	This study	SJC338
tiam-1(tm1556) I; pxf-1(gk955083) IV; Punc-129::mCherry::RAB-3(tauIs46)	This study	SJC406
tiam-1(ok772) I; Punc-129::mCherry::RAB-3(tauIs46)	This study	SJC512
tiam-1(ok772) I; pxf-1(gk955083) IV; Punc-129::mCherry::RAB-3(tauIs46)	This study	SJC513
Punc-129::mCherry::RAB-3(tauIs46); Punc-17b::mGFP::utrophin-CH(bluEx30)	(Lamb et al., 2022)	SJC316
rac-2(ok326) IV; Punc-129::mCherry::RAB-3(tauIs46); Punc-17b::mGFP::utrophin-CH(bluEx30)	This study	SJC358
Punc-129::mCherry::RAB-3(tauIs46); Punc-17b::rap-1(WT)::mGFP (bluEx143)	This study	SJC690
pxf-1(gk955083) IV; Punc-129::mCherry::RAB-3(tauIs46); Punc-17b::rap-1(WT)::mGFP (bluEx143)	This study	SJC691
Punc-129::mCherry::RAB-3(tauIs46); Punc-17b::rap-1(WT)::mGFP (bluEx144)	This study	SJC692
pxf-1(gk955083) IV; Punc-129::mCherry::RAB-3(tauIs46); Punc-17b::rap-1(WT)::mGFP (bluEx144)	This study	SJC693
Punc-129::mCherry::RAB-3(tauIs46); Punc-17b::rap-1(G12V)::mGFP (bluEx146)	This study	SJC694
pxf-1(gk955083) IV; Punc-129::mCherry::RAB-3(tauIs46); Punc-17b::rap-1(G12V)::mGFP (bluEx146)	This study	SJC695
Punc-129::mCherry::RAB-3(tauIs46); Punc-17b::rap-1(G12V)::mGFP (bluEx147)	This study	SJC696
Punc-129::mCherry::RAB-3(tauIs46); Punc-17b::rac-2(WT)::mGFP (bluEx166)	This study	SJC704
pxf-1(gk955083) IV; Punc-129::mCherry::RAB-3(tauIs46); Punc-17b::rac-2(WT)::mGFP (bluEx166)	This study	SJC705
Punc-129::mCherry::RAB-3(tauIs46); Punc-17b::rac-2(WT)::mGFP (bluEx168)	This study	SJC706
pxf-1(gk955083) IV; Punc-129::mCherry::RAB-3(tauIs46); Punc-17b::rac-2(WT)::mGFP (bluEx168)	This study	SJC707
Punc-129::mCherry::RAB-3(tauIs46); Punc-17b::rac-2(G12V)::mGFP (bluEx169)	This study	SJC708
pxf-1(gk955083) IV; Punc-129::mCherry::RAB-3(tauIs46); Punc-	This study	SJC709

Development • Supplementary information

17b::rac-2(G12V)::mGFP (bluEx169)		
Punc-129::mCherry::RAB-3(tauIs46); Punc-17b::rac-2(G12V)::mGFP (bluEx171)	This study	SJC710
pxf-1(gk955083) IV; Punc-129::mCherry::RAB-3(tauIs46); Punc-17b::rac-2(G12V)::mGFP (bluEx171)	This study	SJC711
Punc-17b::mCherry::RBD::T2A::rac-2::mGFP(bluEx71)	This study	SJC380
Punc-17b::mCherry::RBD::T2A::rac-2::mGFP(bluEx72)	This study	SJC381
tiam-1(tm1556) I; Punc-17b::mCherry::RBD::T2A::rac-2::mGFP(bluEx71)	This study	SJC431
tiam-1(tm1556) I; Punc-17b::mCherry::RBD::T2A::rac-2::mGFP(bluEx72)	This study	SJC432
pxf-1(gk955083) IV; Punc-17b::mCherry::RBD::T2A::rac-2::mGFP(bluEx71)	This study	SJC547
pxf-1(gk955083) IV; Punc-17b::mCherry::RBD::T2A::rac-2::mGFP(bluEx72)	This study	SJC515
Punc-17b::tiam-1::mGFP + Punc-17b::mCherry(bluEx148)	This study	SJC639
Punc-17b::tiam-1::mGFP + Punc-17b::mCherry(bluEx149)	This study	SJC640
Punc-17b::tiam-1::mGFP + Punc-17b::mCherry(bluEx150)	This study	SJC641
Punc-17b::tiam-1::mGFP + Punc-17b::rap-1::mCherry (bluEx152)	This study	SJC643
Punc-17b::tiam-1::mGFP + Punc-17b::rap-1::mCherry (bluEx153)	This study	SJC644
Punc-17b::tiam-1::mGFP + Punc-17b::rap-1::mCherry (bluEx154)	This study	SJC645
Punc-129::mCherry::RAB-3(tauIs46); Punc-17b::rap-1(WT)::mGFP (bluEx143)	This study	SJC723
rac-2(ok326) IV; Punc-129::mCherry::RAB-3(tauIs46); Punc-17b::rap-1(WT)::mGFP (bluEx143)	This study	SJC724
Punc-129::mCherry::RAB-3(tauIs46); Punc-17b::rap-1(G12V)::mGFP (bluEx146)	This study	SJC725
rac-2(ok326) IV; Punc-129::mCherry::RAB-3(tauIs46); Punc-17b::rap-1(G12V)::mGFP (bluEx146)	This study	SJC726

Development • Supplementary information

Table S2. Oligonucleotides used in this study

Oligonucleotide sequence (5'->3')	Source	Primer
tgtaaaacgacggccagt	Integrated DNA Technologies	M13-fwd
caggaaacagctatgaccatg	Integrated DNA Technologies	M13-rev
tgaatgaaccctctgaaagtgg	Integrated DNA Technologies	oRL4
ggtgagcccagtacgaattg	Integrated DNA Technologies	oRL5
gagatttattggccgtccgtcg	Integrated DNA Technologies	oRL6
gggtggcgacagagatttgc	Integrated DNA Technologies	oSC48
accctctctgccatactagc	Integrated DNA Technologies	oSC49
gaagtcgacggccagcaatg	Integrated DNA Technologies	oSC236
attgcacgggttcgagcatg	Integrated DNA Technologies	oSC237
ggtagtggaggtgtcggaaa	Integrated DNA Technologies	oRL21
atgaaacgtctgttgactggtg	Integrated DNA Technologies	oRL22
caaggtggaaccggacaatg	Integrated DNA Technologies	oRL23
ttgctcagattagggtgagc	Integrated DNA Technologies	oBM62
acactcgcattacagaatcctg	Integrated DNA Technologies	oBM63
agagtgaaacacgaacacaacg	Integrated DNA Technologies	oBM64
gacaaatggacgtcttggactc	Integrated DNA Technologies	oBM65
tttcgggctccgaaagtag	Integrated DNA Technologies	oBM66
cgaattcgcccttggactagtcatatgggtaccatgcgggagtataag attgttgtg	Integrated DNA Technologies	oSC188
ccagatcctccggaaccactggtacccatgatgacacacgagcagc	Integrated DNA Technologies	oSC189
cgcccttggactagtcatatgggtaccatgcaagcaatcaaatgtgtc gtc	Integrated DNA Technologies	oSC304
ccagatcctccggaaccactggtacccaccgtgcaattgctctttttg	Integrated DNA Technologies	oSC305

Development • Supplementary information
